# Electrospinning onto Insulating Substrates by Controlling Surface Wettability and Humidity

**DOI:** 10.1186/s11671-017-2380-6

**Published:** 2017-11-28

**Authors:** WooSeok Choi, Geon Hwee Kim, Jung Hwal Shin, Geunbae Lim, Taechang An

**Affiliations:** 1 Department of Mechanical Engineering, Korea National University of Transportation, Chungju, Chungcheongbuk-do 380-702 Republic of Korea; 2 Department of Mechanical Engineering, Pohang University of Science and Technology (POSTECH), Pohang, 790-784 Republic of Korea; 3 Biomedical Engineering, School of Life Sciences, Ulsan National Institute of Science and Technology (UNIST), 50, UNIST-gil, Ulsan, 44919 Republic of Korea; 4 Department of Mechanical Design Engineering, Andong National University, Kyungbuk, 760-749 Republic of Korea

**Keywords:** Electrospinning, Nanofibers, Surface wettability, Thin film water, Insulator substrate

## Abstract

We report a simple method for electrospinning polymers onto flexible, insulating substrates by controlling the wettability of the substrate surface. Water molecules were adsorbed onto the surface of a hydrophilic polymer substrate by increasing the local humidity around the substrate. The adsorbed water was used as the ground electrode for electrospinning. The electrospun fibers were deposited only onto hydrophilic areas of the substrate, allowing for patterning through wettability control. Direct writing of polymer fiber was also possible through near-field electrospinning onto a hydrophilic surface.

**Electronic supplementary material:**

The online version of this article (10.1186/s11671-017-2380-6) contains supplementary material, which is available to authorized users.

## Background

Electrospinning is a technique used to produce continuous fibers, with diameters of several hundred nanometers, using an electric field. Electrospinning is relatively inexpensive and has been applied to a wide variety of applications and materials [1–4]. The electrospinning setup consists primarily of three parts: a high-voltage source, a spinneret, and a collector. The collector is generally a conductive substrate, such as a metal, that functions as the ground electrode and helps form a stable electric field in the spinneret. When non-conductive substrates are used as collectors, conductive ground electrodes must be placed on the substrate surface [4, 5].

Many industrial applications of electrospun nanofibers require their deposition onto insulating substrates, such as flexible polymers [6, 7]. Cho et al. [6] demonstrated the deposition of electrospun nanofibers onto thin, flexible insulator layers on an electrode. Electrospun nanofibers deposited under such circumstances will follow or align with the underlying electrodes. Min et al. [8] produced patterned organic semiconducting nanowires on a polymer substrate using near-field electrospinning. In both cases, electrospinning onto the polymer substrate was only possible if the insulating layer was thin enough (less than 100 μm) to maintain a high electric field. Zheng et al. [7] reported electrospinning onto an insulating polymer substrate (polyethylene terephthalate) using an AC pulse-modulated electrohydrodynamic method. This method is capable of electrospinning onto polymer substrates regardless of substrate thickness, but requires the application of a relatively complex AC electric field. While the aforementioned studies have demonstrated feasibility, electrospinning onto non-conductive surfaces has not attained widespread use in industrial applications.

Here, we present a novel method for electrospinning fibers onto insulating substrates that overcomes the limitations of previous work. Electrospinning has been demonstrated using a liquid electrolyte as the collector electrode [9–12]. Also note that, at an appropriately high humidity, water molecules will adsorb to a hydrophilic surface and begin to conduct electricity at approximately one monolayer [13]. If the proper humidity is maintained around an insulating substrate with a hydrophilic surface, then water molecules adsorbed on the surface can serve as an electrode layer, allowing the deposition of electrospun fibers. Unlike previous studies, this method is independent of substrate thickness because it relies only on the surface characteristics of the substrate in the surrounding environment. Moreover, it is compatible with conventional electrospinning techniques, requiring only humidity control.

## Methods

### Preparation of Polymer Substrate with a Hydrophilic

In this experiment, a 500-μm acrylic substrate with an originally hydrophobic surface was used as the collector. Oxygen plasma treatment (CUTE, Femto Science, Korea) for 30 s of the acrylic substrate resulted in a hydrophilic surface populated with silanol groups (SiOH) [14]. This reaction was confirmed by a change in water contact angle from 81.3° on pristine acrylic to 36.7° after plasma treatment (Additional file 1: Figure S1b–d). Regions of the acrylic substrate were selectively made hydrophilic by applying a stencil mask prior to plasma treatment (Additional file 1: Figure S1a).

### Preparations for Electrospinning

**Fig. 1:**
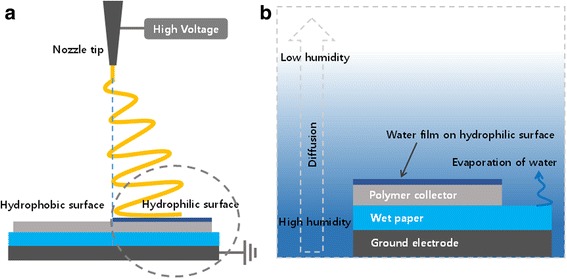
Schematic diagram showing (**a**) the electrospinning process on a polymer substrate with local humidity control, and (**b**) is details of boundary region of (**a**)

Electrospinning was performed at room temperature and moderate humidity (relative humidity 40~50%) with 10 wt% polyurethane (PU) (Pellethane 2363-80AE; Lubrizol, USA) dissolved in a mixture (80/20, *v*/*v*) of tetrahydrofuran (THF) and dimethylformamide (DMF). To compare the effects of surface hydrophobicity, an acrylic substrate with both hydrophilic and hydrophobic surfaces was placed on the ground electrode and used as a collector during electrospinning (Fig. 1a).

### Local Humidity Control

To increase the humidity in the immediate vicinity of the polymer substrate, a wet paper was placed between the polymer substrate and the ground electrode (Fig. 1b). The humidity was relatively high only around the polymer substrate due to the low diffusivity of water vapor. The humidity around the electrospinning syringe tip was about 50%, while the humidity around the polymer substrate was about 70% (Additional file 1: Figure S2). It has been shown that water molecule adsorption onto the surface of hydrophilic polymers increases rapidly when the relative humidity exceeds 50% [15].

## Results and Discussion

### The Force Acting on CNTs at the Liquid–Air Interface

We investigated two modes of electrospinning: a tip-to-electrode distance of 8 cm and applying 13 kV DC voltage with a fixed tip (far-field electrospinning), and a tip-to-electrode distance of 1 cm and applying 2 kV DC voltage with a moving tip (near-field electrospinning).

**Fig. 2:**
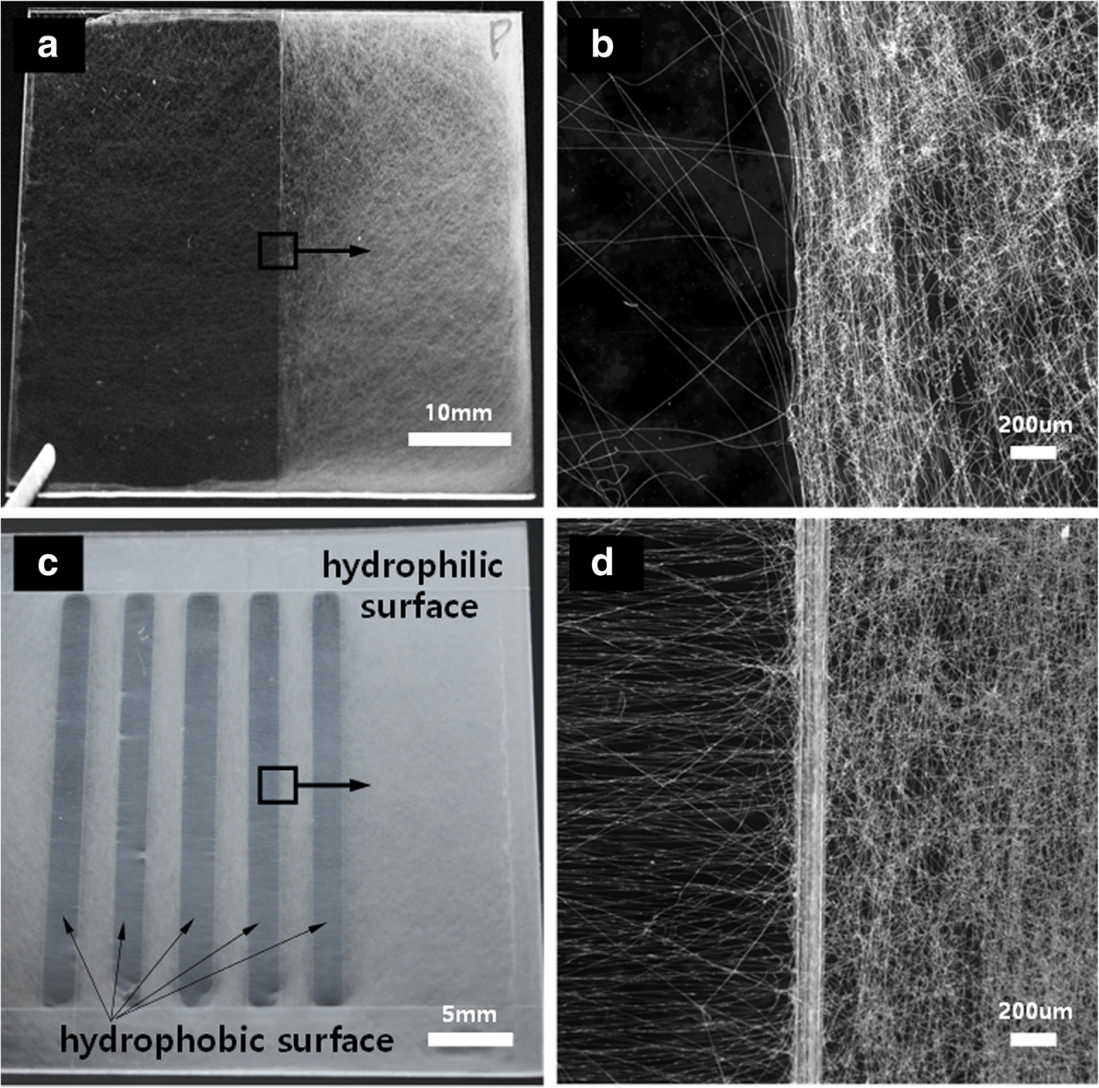
Images of far-field electrospun films on surfaces with different wettabilities. **a**, **c** Digital camera photographs. **b**, **d** Digital micrographs of the boundary region of **a** and **c**, respectively

Far-field electrospinning was performed by first placing the polymer substrate on the ground electrode. Electrospinning did not occur on hydrophobic areas of the substrate. Instead, the polymer solution would form a droplet at the end of the tip, eventually falling due to gravity. In contrast, when the hydrophilized polymer substrate was placed on the electrode, electrospun fibers were deposited on the substrate surface, as is observed with conventional electrospinning using conductive substrates. Electrospun fibers were then deposited onto a dual substrate having both hydrophobic and hydrophilic surfaces. Figure 2 shows digital camera photographs and micrographs of electrospun nanofibers on the dual substrate. Most of the fibers were deposited on the hydrophilic surface. In Fig. 2a, b, the right and left halves of the polymer surface are hydrophilic and hydrophobic, respectively. The syringe tip was fixed at the center of the substrate. Water vapor from the air had adsorbed only on the hydrophilic surface, acting as an electrode. An electric field was formed between the tip and the water when a high voltage was applied for electrospinning. In contrast, the hydrophobic surface of the pristine acrylic substrate prevented the formation of an electric field between the tip and ground electrode. Electrospinning is a phenomenon in which a charged solution exits a syringe tip via a repulsive electrostatic force. The droplet of polymer solution that exits the jet is therefore charged. The charged polymer solution experiences the electrostatic force and moves toward the hydrophilic surface. For the same reason, electrospinning did not occur on the hydrophobic region of the electrode. The electrospun fibers deposited at the edge of the hydrophobic domain in Fig. 2a are presumed to be due to the influence of the electrode exposed to the outside of the polymer substrate. In Fig. 2c, d, five parallel bars of polymer substrate and the rest were hydrophobic and hydrophilic, respectively. The width and spacing of the bars were 2 mm. Electrospun fibers deposited on the hydrophobic surface were aligned with their longitudinal axes oriented perpendicular to the boundary of hydrophilic and hydrophobic surfaces. But the electrospun fibers on the hydrophilic surface were randomly disordered. This is consistent with well-known results in conventional electrospinning based on metal electrodes [16].

**Fig. 3:**
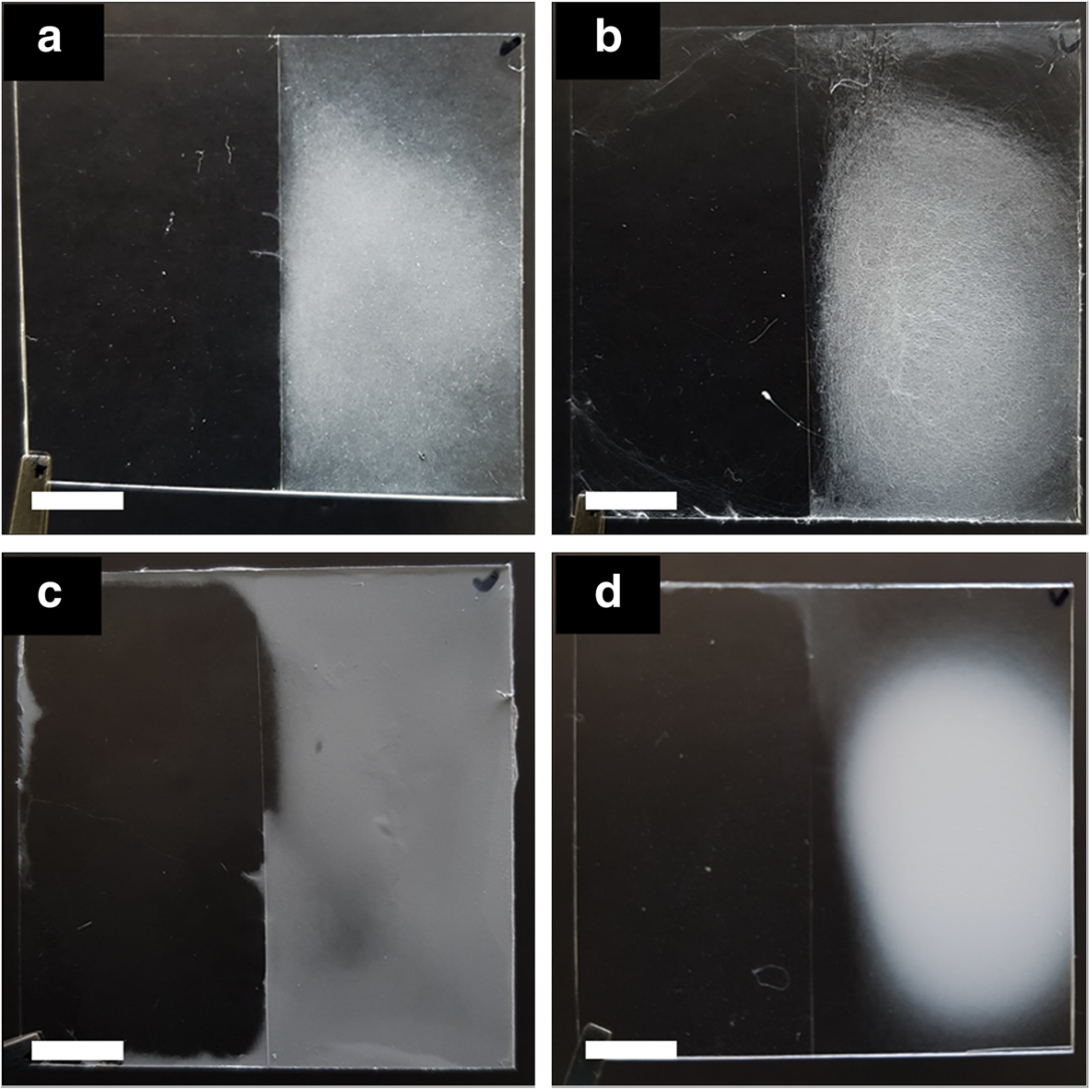
Images of electrospun fiber on polymer substrate with hydrophilic (right) and hydrophobic (left) surface. **a** PCL. **b** PS. **c** Cellulose acetate. **d** PVDF (scale bar: 10 mm)

In order to verify the versatility, electrospinning was carried out for four kinds of polymers: PCL (polycaprolactone), PS (polystyrene), CA (cellulose acetate), and PVDF (polyvinylidene fluoride). PCL (15 wt%, Sigma-Aldrich) was dissolved in a mixture (20/80, *v*/*v*) of THF and DMF, PS (10 wt%, Sigma-Aldrich) was dissolved in a mixture (80/20, *v*/*v*) of THF and DMF, CA (10 wt%, Sigma-Aldrich) was dissolved in a mixture (1/1, *v*/*v*) of acetone and dimethylacetamide (DMAc), and PVDF (15 wt%, Sigma-Aldrich) was dissolved in DMF at 60 °C, respectively. In Fig. 3, four different electrospun fibers are deposited on the surface of hydrophilic surface like PU electrospun fibers.

**Fig. 4:**
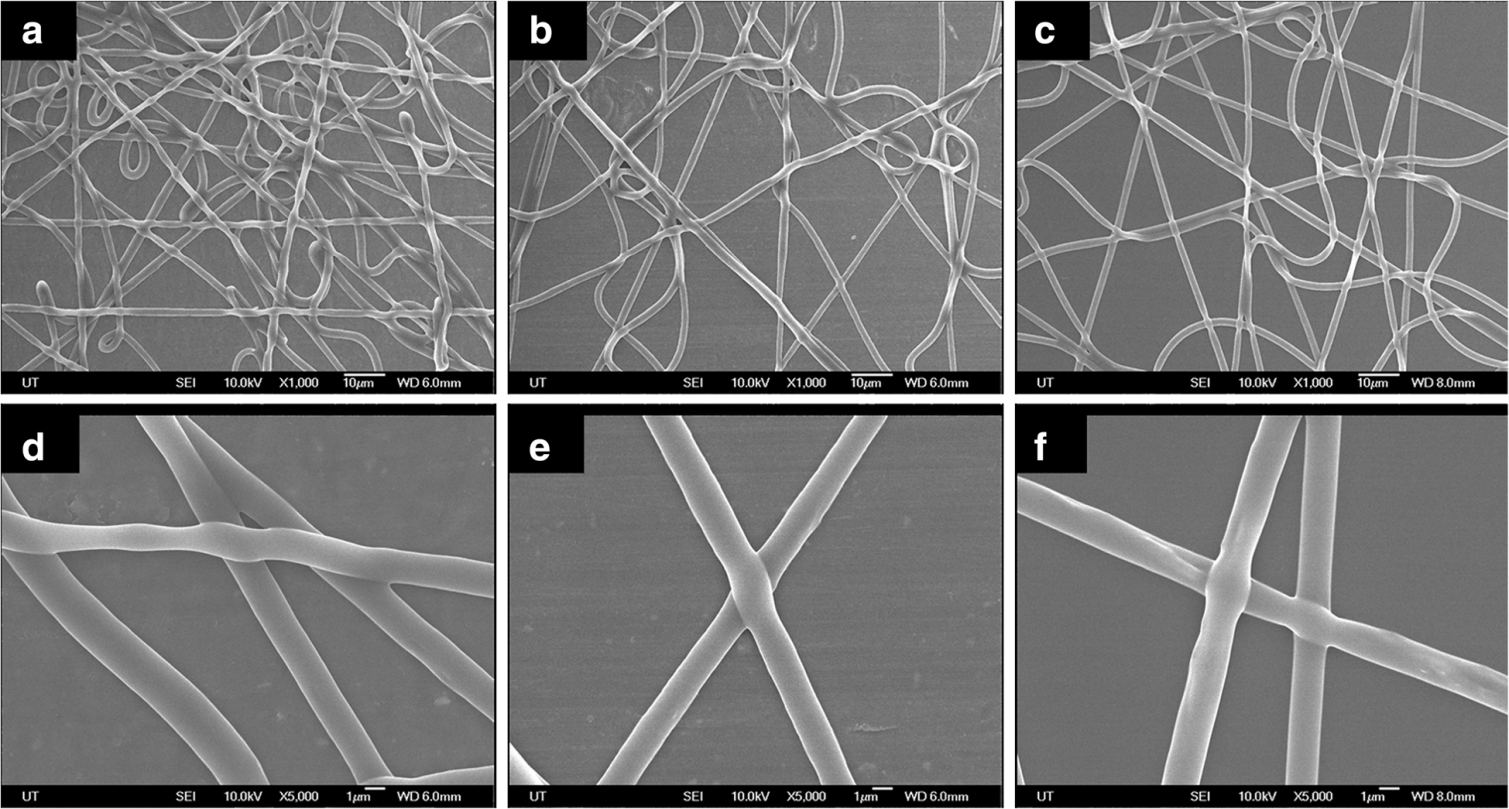
SEM images of electrospun fiber under different conditions with a tip-to-electrode distance of 8 cm and applying 12 kV DC voltage. **a**, **d** Conventional electrospinning—metal electrode without local humidity control. **b**, **e** Metal electrode with local humidity control. **c**, **f** Hydrophilic surface polymer substrate with local humidity control

The morphology of the electrospun fiber on the polymer substrate was compared with the conventional electrospinning and fiber on the metal electrode with locally humidity control. Figure 4 shows the SEM image of PU electrospun fiber onto metal electrode with and without locally humidity control and polymer substrate with locally humidity control. The morphology of electrospun fibers was similar in all three cases. It is presumed that strong volatile solvents evaporate sufficiently because humidity maintains low around the syringe.

**Fig. 5:**
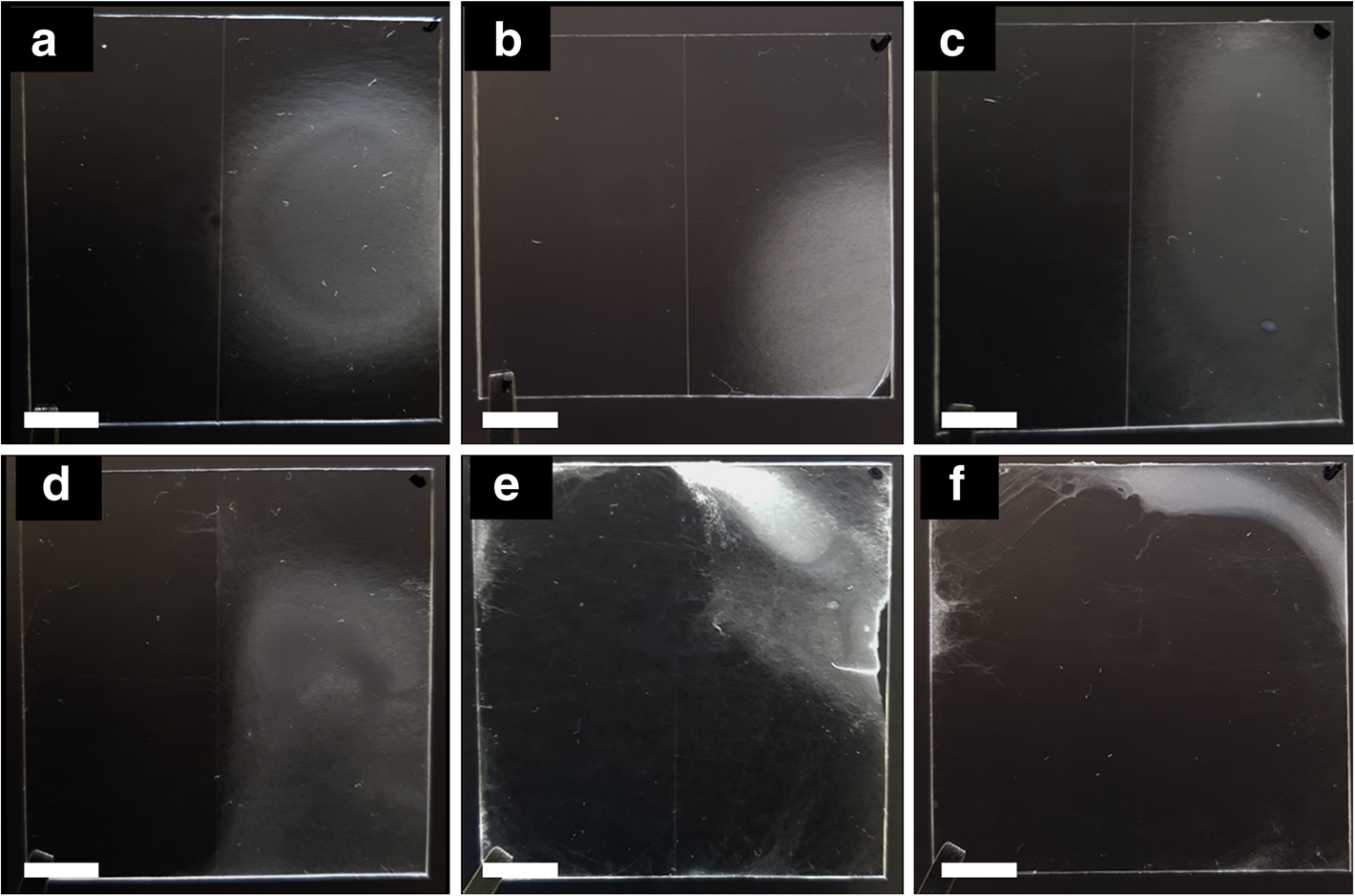
Images of PU electrospun fiber on polymer substrate with hydrophilic (right) and hydrophobic (left) surface according to applied DC voltage for 2 min. **a** 6 kV. **b** 8 kV. **c** 10 kV. **d** 12 kV. **e** 14 kV. **f** 16 kV (scale bar: 10 mm)

The intensity of the electric field is one of the important factors for altering the pattern of the electrospun fibers. Figure 5 shows the pattern of electrospun fiber onto a polymer substrate with hydrophilic (right) and hydrophobic (left) surface where applied voltage was changed from 6 to 16 kV at a tip-to-electrode distance of 8 cm. It is known that as the electric field increases, the loops of polymer jet become larger as the bending instability increases [17, 18]. As the loops of polymer jet grow, electrospun fibers deposit on the electrode exposed to the outside of the polymer substrate. Therefore, electrospun fibers deposit on the hydrophobic surface of polymer substrate between the electrode and the hydrophilic surface. On the other hand, when the loops of polymer jet are small, most of the electrospun fibers deposit on the hydrophilic surface of the polymer substrate located vertically below the syringe tip.

**Fig. 6:**
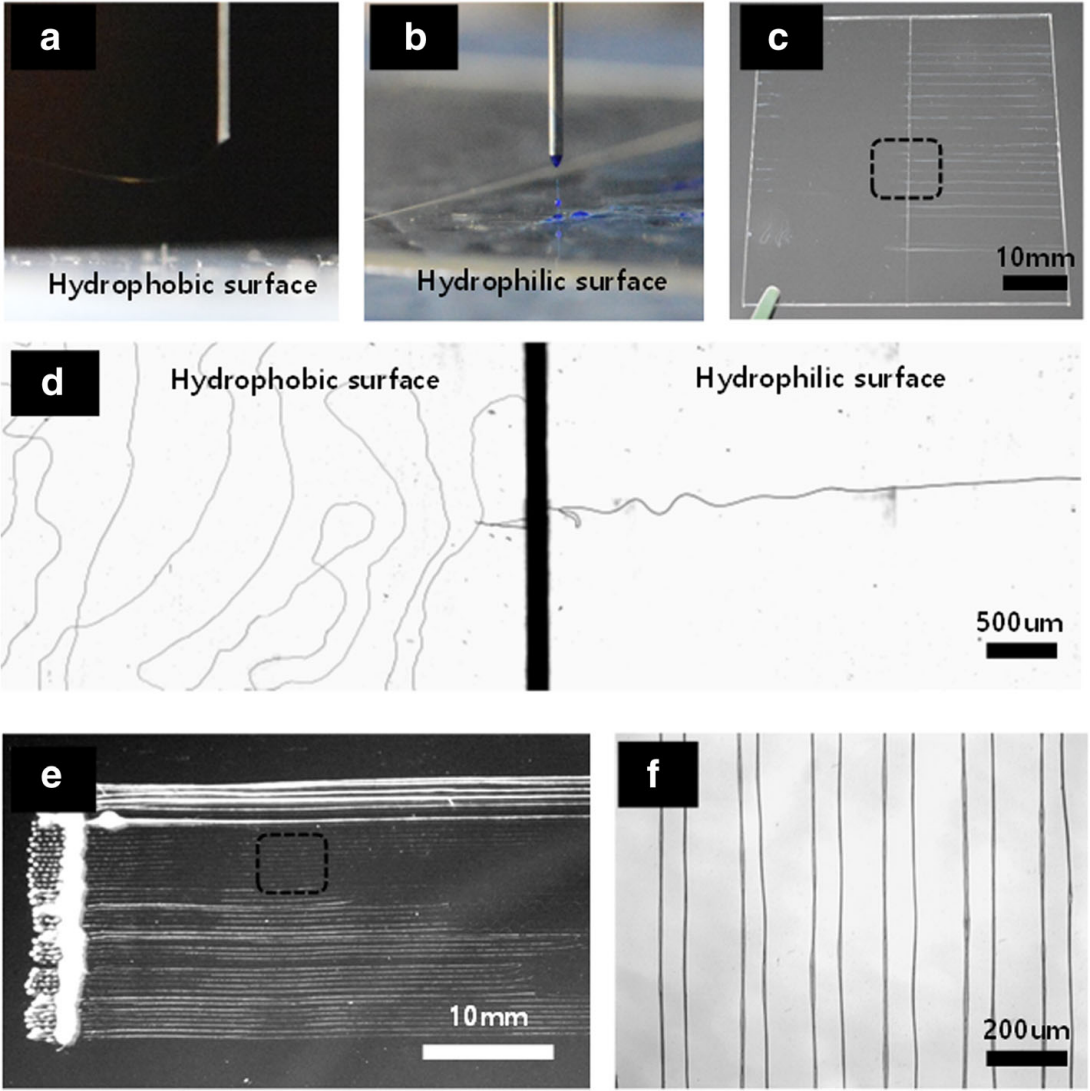
Images of near-field electrospun films on **a** a hydrophobic surface and **b** a hydrophilic surface. Images of polymer fibers written directly onto a polymer substrate with a hydrophobic surface (left) and a hydrophilic surface (right); **c** a digital camera photograph and **d** a digital micrograph. Images of electrospun polymer fibers written directly on a hydrophilic surface; **e** a digital camera photograph and **f** a digital micrograph

Near-field electrospinning was performed at a tip-to-substrate distance of 1 cm and the tip was moved at a rate of 100 mm/s. Figure 6a, b compares direct-patterned polymer nanofibers on a conductive electrode and a hydrophilic polymer substrate. When presented with a hydrophobic region on the electrode, fibers were emitted toward the exposed electrode. Conversely, fibers were emitted directly toward the hydrophilized polymer substrate. Charges in a droplet of polymer solution are unable to escape if the droplet falls onto an insulating surface. Thus, the charge of this initially deposited polymer layer will repel incoming electrospun droplets [19]. Figure 6c, d shows the result of polymer fibers being written directly onto a polymer substrate having both hydrophobic and hydrophilic surfaces. The vertical line in the image is the border between the hydrophilic (left) and hydrophobic (right) regions. Fibers on the hydrophilic surface were drawn along the tip path in a straight line and were similar in form to fibers made via conventional near-field electrospinning. In contrast, fibers on the hydrophobic surface were unstable and exhibited twisted or curved shapes. Fibers on the hydrophilic surface were placed by inertia resulting from the moving tip, as it moved from the hydrophilic region. Polymer fibers falling in such a way were highly unstable due to the lack of an electric field on the hydrophobic surface. Figure 6e shows fibers resulting from the direct writing of polymer lines onto the hydrophilic polymer substrate. Note that Fig. 6f is an enlargement of Fig. 6e. These data confirm that polymer patterns can be drawn directly onto the surface of an insulator with a hydrophilic surface as they would be drawn on an electrode surface.

## Conclusions

We introduced a novel method for electrospinning onto an insulating substrate regardless of substrate thickness. Plasma treatment of an acrylic substrate produces a hydrophilic surface. In an appropriately high-humidity environment, water molecules adsorb to form a thin layer that acts as a ground electrode. Electrospun nanofibers were deposited on a flexible polymer substrate using this method and there was no significant difference from the morphology of electrospun fiber from conventional electrospinning. It was also shown that polymer fibers could be written directly on hydrophilic surfaces of hydrophobic substrates using near-field electrospinning. Increasing the local humidity around the polymer substrate enabled electrospinning onto the insulator surface. This interesting result contrasts with the general assumption that electrospinning should be performed at low humidity. Specific regions of a polymer substrate can be defined for electrospun fiber deposition by selectively controlling the wettability of the substrate. Therefore, fiber patterns are possible without the relatively complex and expensive processes, such as microelectromechanical system (MEMS)-based techniques, currently used to fabricate micropatterned electrodes. Moreover, we believe that electrospinning using conductive materials such as carbon nanotubes or conducting polymers may be applicable to fabricating electrodes on flexible substrates that can be used in wearable devices.

## Additional file

Additional file 1: Figure S1. (a) Schematic diagram shows the electrospinning process and selective oxygen plasma treatment. Contact angles were measured on a (b) pristine polymer substrate and (c) polymer substrate following oxygen plasma treatment. (d) A graph of plasma treatment time versus contact angle of polymer substrate. **Figure S2.** Relative humidity near the polymer substrate and syringe tip. (DOC 464 kb)
