# Supplementary material for: Electrospinning onto Insulating Substrates by Controlling Surface Wettability and Humidity
Source: Nanoscale Res Lett. 2017 Nov 28;12:610. doi: 10.1186/s11671-017-2380-6 (PMC5705525; doi:10.1186/s11671-017-2380-6)
Supplement: Additional file 1: Figure S1. — (a) Schematic diagram shows the electrospinning process and selective oxygen plasma treatment. Contact angles were measured on a (b) pristine polymer substrate and (c) polymer substrate following oxygen plasma treatment. (d) A graph of plasma treatment time versus contact angle of polymer substrate. Figure S2. Relative humidity near the polymer substrate and syringe tip. (DOC 464 kb) [file 11671_2017_2380_MOESM1_ESM.doc]

**Additional file**


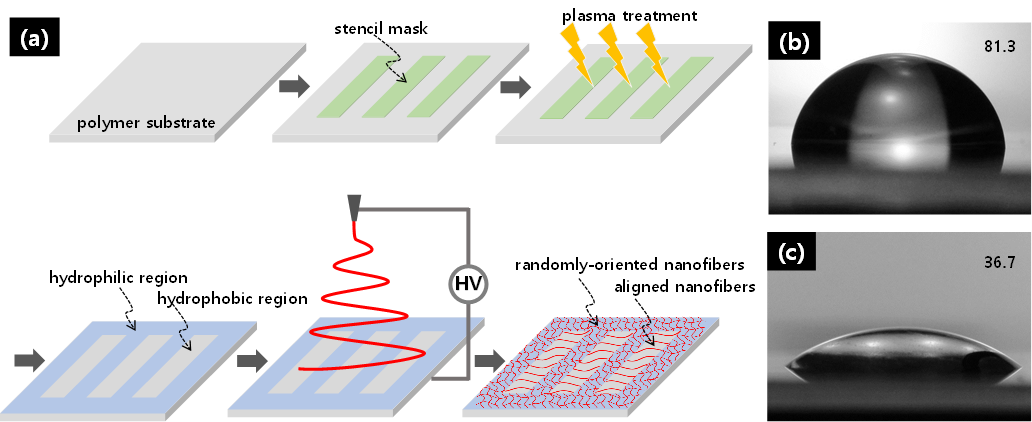


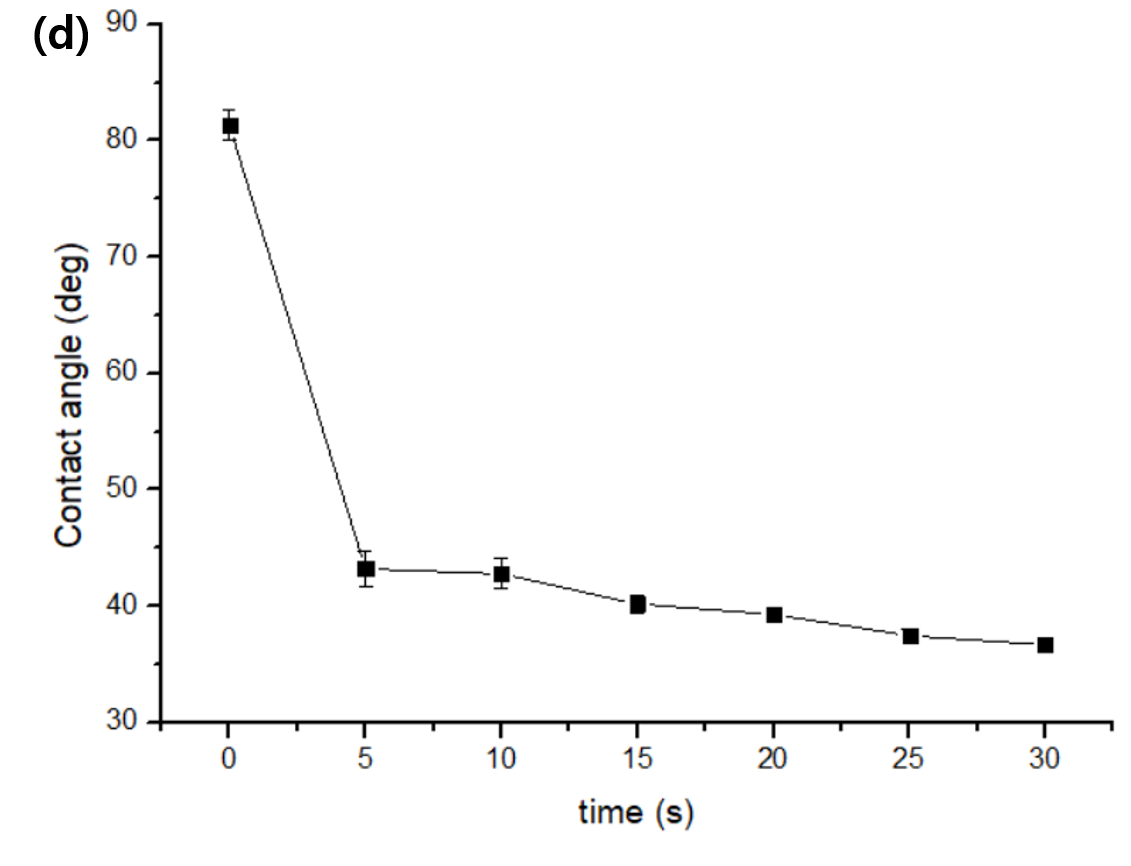


**Figure S1.** (a) Schematic diagram shows the electrospinning process and selective oxygen plasma treatment. Contact angles were measured on a (b) pristine polymer substrate and (c) polymer substrate following oxygen plasma treatment. (d) A graph of plasma treatment time versus contact angle of polymer substrate.


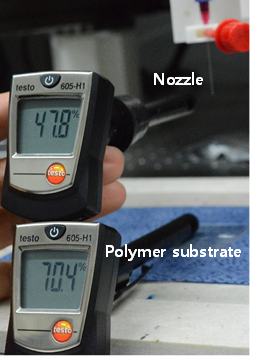


**Figure S2.** Relative humidity near the polymer substrate and syringe tip.
